# A machine learning evaluation of patient characteristics associated with prescribing of guideline-directed medical therapy for heart failure

**DOI:** 10.3389/fcvm.2023.1169574

**Published:** 2023-06-21

**Authors:** Rachel Kim, Krithika Suresh, Michael A. Rosenberg, Malinda S. Tan, Daniel C. Malone, Larry A. Allen, David P. Kao, Heather D. Anderson, Premanand Tiwari, Katy E. Trinkley

**Affiliations:** ^1^ School of Medicine, University of Colorado Medical Campus, Aurora, CO, United States; ^2^ Department of Biostatistics and Informatics, Colorado School of Public Health, Aurora, CO, United States; ^3^ Department of Pharmacotherapy, University of Utah, Salt Lake City, UT, United States; ^4^ Adult and Child Consortium for Outcomes Research and Delivery Science (ACCORDS), University of Colorado Anschutz Medical Campus, Aurora, CO, United States; ^5^ Department of Clinical Informatics, UCHealth, Aurora, CO, United States; ^6^ Department of Clinical Pharmacy, University of Colorado Anschutz Medical Campus Skaggs School of Pharmacy and Pharmaceutical Sciences, Aurora, CO, United States

**Keywords:** heart failure, electronic health record, machine learning, population health, prescribing

## Abstract

**Introduction/background:**

Patients with heart failure and reduced ejection fraction (HFrEF) are consistently underprescribed guideline-directed medications. Although many barriers to prescribing are known, identification of these barriers has relied on traditional *a priori* hypotheses or qualitative methods. Machine learning can overcome many limitations of traditional methods to capture complex relationships in data and lead to a more comprehensive understanding of the underpinnings driving underprescribing. Here, we used machine learning methods and routinely available electronic health record data to identify predictors of prescribing.

**Methods:**

We evaluated the predictive performance of machine learning algorithms to predict prescription of four types of medications for adults with HFrEF: angiotensin converting enzyme inhibitor/angiotensin receptor blocker (ACE/ARB), angiotensin receptor-neprilysin inhibitor (ARNI), evidence-based beta blocker (BB), or mineralocorticoid receptor antagonist (MRA). The models with the best predictive performance were used to identify the top 20 characteristics associated with prescribing each medication type. Shapley values were used to provide insight into the importance and direction of the predictor relationships with medication prescribing.

**Results:**

For 3,832 patients meeting the inclusion criteria, 70% were prescribed an ACE/ARB, 8% an ARNI, 75% a BB, and 40% an MRA. The best-predicting model for each medication type was a random forest (area under the curve: 0.788–0.821; Brier score: 0.063–0.185). Across all medications, top predictors of prescribing included prescription of other evidence-based medications and younger age. Unique to prescribing an ARNI, the top predictors included lack of diagnoses of chronic kidney disease, chronic obstructive pulmonary disease, or hypotension, as well as being in a relationship, nontobacco use, and alcohol use.

**Discussion/conclusions:**

We identified multiple predictors of prescribing for HFrEF medications that are being used to strategically design interventions to address barriers to prescribing and to inform further investigations. The machine learning approach used in this study to identify predictors of suboptimal prescribing can also be used by other health systems to identify and address locally relevant gaps and solutions to prescribing.

## 1. Introduction

Most patients with HFrEF have significant gaps in the prescribing of evidence-based medications (1–8). In the absence of intolerance or contraindications, many barriers to prescribing these medications have been described. Barriers include clinical inertia and patient characteristics such as certain thresholds for vitals or laboratory values, e.g., blood pressure and potassium (5, 8–11).

Given the large potential benefits of treatment (1–8), it is critical to identify all factors associated with underprescribing. Known barriers to HFrEF prescribing have been identified using traditional quantitative and qualitative methods. However, traditional quantitative methods require an *a priori* signal to evaluate, while qualitative methods require clinician awareness of factors that may influence their prescribing behaviors. These traditional methods are also limited in their ability to comprehensively consider the complexity of factors that can dynamically influence prescribing, leaving the possibility of some barriers being undiscovered. A comprehensive understanding of the underpinnings driving underprescribing can lead to the strategic design of interventions to address barriers (12).

Machine learning (ML) methods are a hypothesis-free means to identify factors that influence prescribing for HFrEF when the relationships between predictors and the outcome is unknown. ML may offer advantages over traditional approaches when applied to healthcare data, in particular mitigating the need to prespecify variable relationships and handling complex interactions and nonlinear variable effects (13–16). Applying such approaches to real-world electronic health record (EHR) data also facilitates a pragmatic evaluation of predictors in a real-world clinical context where such interactions and nonlinear variable effects are important to account for.

In this single-center study, we used ML and regression-based approaches to identify important predictors of prescribing evidence-based medications for HFrEF that are routinely available within the EHR. We compared penalized regression, decision trees, and random forests for predicting prescribing of evidence-based medications for HFrEF. For the best-performing model, we ascertained the most important predictors of prescribing that could then be used by the health system to design interventions or inform areas in need of further investigation.

## 2. Materials and methods

### 2.1. Study population

This study was conducted at the UCHealth system, which includes three large regional centers (North, Metro, and South) comprised of 12 hospitals, 900 clinics, and over 6,000 physicians who serve academic, rural, suburban, and community settings. The entire system is serviced by a single instance of the Epic EHR software program (Epic Systems, Verona, WI, United States). We collected data on eligible patients from Health Data Compass, our institutional enterprise data warehouse that extracts, integrates, and delivers data from the EHR and medical/medication claims data for patients within the UCHealth system. This protocol was approved by the University of Colorado Multiple Institutional Review Board using deidentified and uniquely encoded datasets, with waiver of informed consent.

Patients included non-deceased adults (age ≥18 years) with prevalent HFrEF documented at any patient visit within the health system on or before December 11, 2018. HFrEF was defined as a most recent ejection fraction value ≤40%. Patients were excluded if they did not have at least one outpatient visit with cardiology or primary care between December 11, 2016, and December 11, 2018. These patients were excluded to maximize representation of patients receiving longitudinal care at UCHealth.

### 2.2. Primary outcome

The outcomes of interest were four types of medications used to treat HFrEF: angiotensin converting enzyme inhibitor/angiotensin receptor blocker (ACE/ARB), angiotensin receptor-neprilysin inhibitor (ARNI), evidence-based beta blocker (BB), or mineralocorticoid receptor antagonists (MRA). Evidence-based BBs included metoprolol succinate, bisoprolol, and carvedilol. A patient was determined to be prescribed a given medication type (yes/no) if at least one medication in that category was ordered or dispensed between December 11, 2017, and December 11, 2019 (latest date of data available). The medication did not need to be prescribed after a patient met eligibility criteria, given that the medications of interest can be used for other indications. This time period was chosen (1) due to limited access to medication dispensing data [via SureScripts (17, 26) integration with the EHR]; (2) to account for the possibility of prescribing a medication with refills that would cover up to a 1-year supply on or before December 11, 2018 (date of study eligibility); and (3) to allow patients with a new diagnosis as of the study eligibility date sufficient time to safely be initiated on the medications (within 1-year post study eligibility). When dispensing data was available through SureScripts, dispenses were evaluated to account for medication orders initiated outside of the health system's EHR. A patient's *index date* associated with a particular medication category is defined as the most recent date that medication was prescribed on or before December 11, 2019. The index date for those who were not prescribed a given medication was defined as the most recent office visit on or before December 11, 2019.

### 2.3. Clinical predictors

Candidate variables were determined by study investigators representing advanced heart failure specialists, general cardiology, electrophysiology, clinical pharmacy, and epidemiology. A total of 126 candidate predictor variables were selected based on availability in structured fields in the EHR and because they are either generally recognized as barriers to prescribing or were determined to be novel predictors to explore. See Supplementary Appendix S1 for a detailed list of candidate variables, which includes patient demographics, social history, insurance type, vitals, diagnoses, procedures, medications, and laboratory values. In some cases, missing values were considered as candidate variables (e.g., missing social security number). The number of unique medications was defined based on 10-digit Generic Product Identifier (GPI) codes that specify the active ingredient including the base formulation. The timing of measuring candidate predictor variables was based on the patient-specific index dates for each medication type (not baseline date of study eligibility) and all available data up to the index date. Baseline characteristics were collected on or before December 11, 2018 (date of study eligibility). Values for predictors that were outside of possible physiologic ranges (e.g., ejection fraction of −99.6) were considered to be erroneous and set to be missing.

### 2.4. Statistical analysis

**Figure 1:**
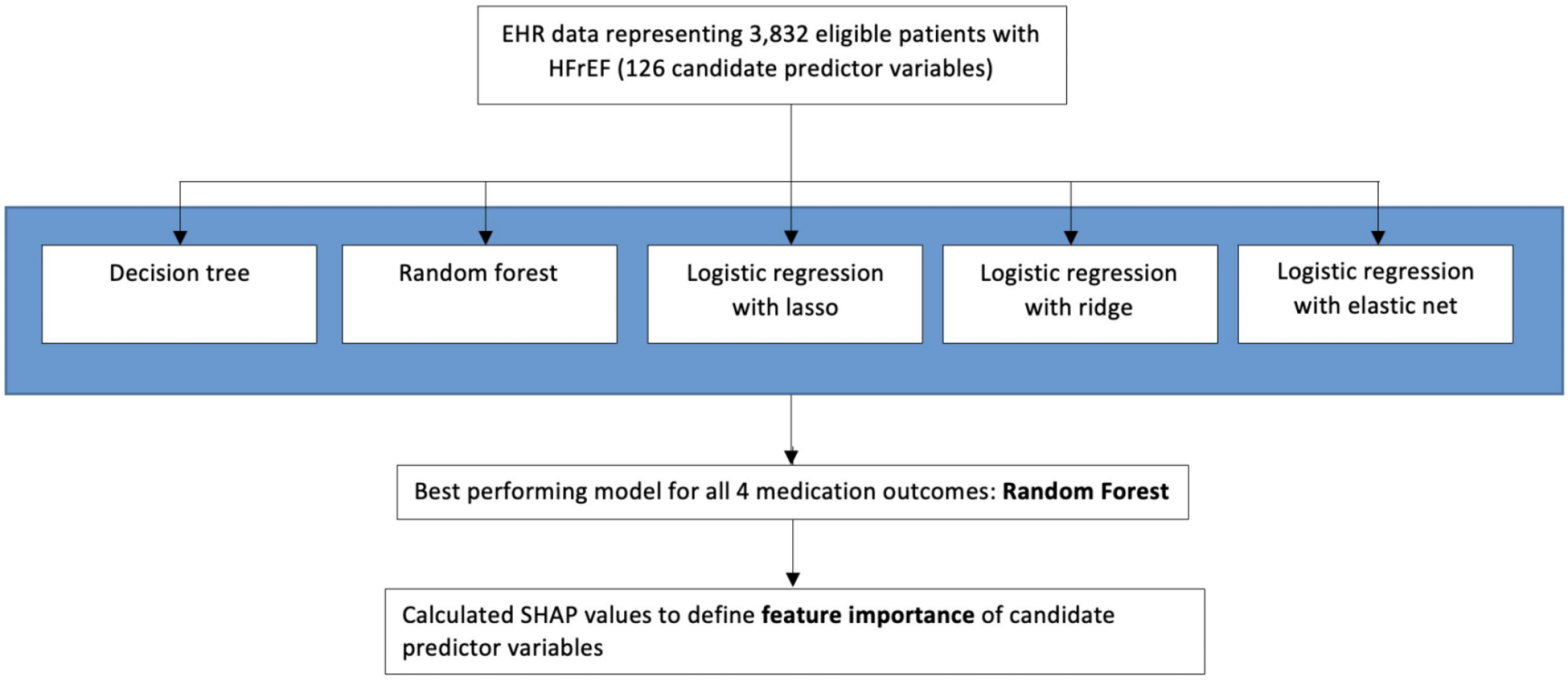
Approach to selecting a predictive model and feature importance of candidate variables.

Patient characteristics were summarized overall and for each medication type. Continuous variables were normalized prior to inclusion in model training. Missing variables were imputed using random forest imputation (18). Four separate analyses were performed to identify top predictors associated with each of the medication outcomes of interest. We trained and evaluated the predictive performance of several predictive models, specifically decision tree, random forest, and logistic regression with lasso, ridge, and elastic net regularization (19–22). Figure 1 provides an overview of the model-building and training process. We performed a 100-fold Monte-Carlo cross-validation using an 80% training and 20% test split. Hyperparameter optimization was performed using a grid search within 5-fold cross-validation on the training set. A manual grid optimization approach was used to ensure that the grid contained the optimal hyperparameters (e.g., if a hyperparameter value was identified on the upper end of the grid range, the grid was expanded to ensure that the overall optimal hyperparameter was not beyond the bounds of the grid space). For outcomes with severe class imbalance (i.e., outcome prevalence of <10%), we oversampled the minority class using the data augmentation method of synthetic minority oversampling (23, 24) in the training set.

We compared the performance of the different predictive models in the test data set. Predictive performance was primarily assessed using area under the receiver operating characteristic curve (AUC), which is a measure of discrimination (i.e., ability to distinguish between high and low probability of prescribing). We compared the differences between the AUCs from the different methods using the DeLong test (25). We additionally report the Brier score, which is a measure of overall performance (26). In this framework, each feature is assigned an importance value for each observation. Shapley Additive Explanation (SHAP) values were reported for the best-performing predictive model to identify the top predictors for prescribing each medication type (27). The mean SHAP value across all observations indicates how much each predictor contributes to the target variable and in what direction. The purpose of developing an ML/regression model is to identify the top predictors of prescribing that jointly predict the outcome. This model is not developed for prospective use for obtaining predictions in clinical practice. The process of identifying the best-performing model based on predictive performance was used to select a model from which to describe the top predictors for prescribing used by that model. A similar approach has been used in other settings to identify clinical predictors of an outcome (28–30).

All analyses were performed in Python 3.7.5 using the packages imblearn 0.0, numpy 1.18.1, pandas 1.1.2, scikit-learn 0.24.2, researchpy 0.3.2, and shap 0.40.0.

## 3. Results

### 3.1. Patient characteristics

A total of 3,832 individuals met the inclusion criteria. The median age of the population was 69 years, 30% were female, and 87.7% were non-Hispanic. Table 1 describes the baseline characteristics of these individuals overall and stratified by medication type. Of these, 69.6% were prescribed an ACE/ARB, 7.5% an ARNI, 74.6% a BB, 39.7% an MRA, and 16.7% were prescribed none of these medications.

**Table 1:** Baseline patient characteristics.

	ACE/ARB (*n *= 2,668)		ARNI (*n *= 289)		EBM/BB (*n *= 2,857)		MRA (*n *= 1,522)		None (*n *= 639)		Overall (*n *= 3,832)	
	Value	Missing	Value	Missing	Value	Missing	Value	Missing	Value	Missing	Value	Missing
Age	68 (21,104)		64 (21, 91)		69 (22, 104)		66 (23, 98)		66 (20, 100)		69 (20, 104)	
Female	784 (29)		65 (22)		839 (29)		431 (28)		216 (34)		1,151 (30)	
Ethnicity												
Hispanic	288 (11)		41 (14)		298 (10)		177 (12)		64 (10)		404 (11)	
Non-Hispanic	2,340 (88)		245 (85)		2,513 (88)		1,325 (87)		557 (87)		3,360 (88)	
Other	40 (2)		3 (1)		46 (2)		20 (1)		18 (3)		68 (2)	
Race						1 (0)				2 (0)		3 (0)
White	1,985 (74)		208 (72)		2,134 (75)		1,098 (72)		487 (76)		2,893 (76)	
Black	305 (11)		38 (13)		321 (11)		197 (13)		57 (9)		399 (10)	
Other	378 (14)		43 (15)		401 (14)		227 (15)		93 (15)		537 (14)	
Insurance												
Commercial	421 (16)		60 (21)		415 (15)		237 (16)		117 (18)		587 (15)	
Medicaid/dual	352 (13)		46 (16)		358 (13)		228 (15)		80 (13)		466 (12)	
Medicare/tricare	1,729 (65)		174 (60)		1,908 (67)		950 (62)		374 (59)		2,515 (66)	
Other	144 (5)		8 (3)		149 (5)		94 (6)		60 (9)		224 (6)	
Self-pay	22 (1)		1 (0)		27 (1)		13 (1)		8 (1)		40 (1)	
Relationship status												
Single	1,136 (43)		107 (37)		1,231 (43)		635 (42)		281 (44)		1,644 (43)	
Relationship	1,429 (54)		170 (59)		1,517 (53)		826 (54)		304 (48)		2,014 (53)	
Other	103 (4)		12 (4)		109 (4)		61 (4)		54 (9)		174 (5)	
Vitals and lab values												
Heart rate	74 (0, 171)	3 (0)	75 (42, 122)		74 (0, 171)	1 (0)	75 (0, 171)	1 (0)	76 (47, 153)	15 (2)	74 (0, 171)	18 (1)
Heart rate >50	24 (1)	3 (0)	4 (1)		25 (1)	1 (0)	16 (1)	1 (0)	5 (1)	15 (2)	33 (1)	18 (1)
SBP, mmHg	119 (62, 204)	3 (0)	114 (72, 186)		118 (62, 204)	1 (0)	116 (62, 198)	1 (0)	122 (74, 196)	15 (2)	120 (62, 204)	18 (1)
SBP >90 mm Hg	73 (3)	3 (0)	12 (4)		73 (3)	1 (0)	62 (4)	1 (0)	14 (2)	15 (2)	104 (3)	18 (1)
DBP, mmHg	72 (0, 137)	3 (0)	70 (0, 137)		71 (0, 137)	1 (0)	70 (0, 137)	1 (0)	74 (0, 120)	15 (2)	72 (0, 137)	18 (1)
DBP >60 mmHg	318 (12)	3 (0)	34 (12)		346 (12)	1 (0)	212 (14)	1 (0)	43 (7)	15 (2)	430 (11)	18 (1)
Ejection fraction	32 (−3, 40)		29 (3, 40)		32 (−23, 40)		30 (1, 40)		34 (−102, 40)		33 (−102, 40)	
Hemoglobin	14 (6, 21)	88 (3)	14 (8, 21)	7 (2)	14 (6, 21)	98 (3)	14 (6, 21)	41 (3)	14 (7, 20)	64 (10)	14 (6, 21)	180 (5)
Sodium	139 (122, 150)	56 (2)	139 (122, 146)	7 (2)	139 (116, 150)	63 (2)	138 (116, 148)	23 (2)	139 (125, 151)	54 (9)	139 (116, 151)	129 (3)
Potassium	4(3, 7)	56 (2)	4 (3, 6)	7 (2)	4 (3, 7)	63 (2)	4 (3, 7)	23 (2)	4 (3, 6)	54 (9)	4 (3, 7)	129 (3)
Hyperkalemia	67 (3)	56 (2)	5 (2)	7 (2)	82 (3)	63 (2)	41 (3)	23 (2)	15 (2)	54 (9)	103 (3)	129 (3)
BUN	21 (2, 149)	56 (2)	20 (5, 87)	7 (2)	21 (2, 140)	63 (2)	21 (3, 134)	23 (2)	19 (4, 124)	54 (9)	21 (2, 149)	129 (3)
eGFR	60 (5, 133)	133 (5)	60 (8, 103)	14 (5)	60 (3, 140)	138 (5)	60 (14, 125)	63 (4)	60 (0, 123)	118 (19)	60 (3, 133)	278 (7)
eGFR		133 (5)		14 (5)		138 (5)		63 (4)		118 (19)		278 (7)
*[0, 30)*	117 (4)		6 (2)		168 (6)		37 (2)		32 (5)		220 (6)	
*[30, 45)*	211 (8)		15 (5)		277 (10)		140 (9)		38 (6)		339 (9)	
*[45, 60)*	406 (15)		43 (15)		466 (16)		252 (17)		81 (13)		583 (15)	
*[60, Inf]*	1,801 (68)		211 (73)		1,808 (63)		1,030 (68)		370 (58)		2,412 (63)	
BNP	232 (2, 3,190)	2,437 (91)	277 (11, 3,180)	261 (90)	258 (2, 3,840)	2,583 (90)	255 (11, 3,180)	1,378 (91)	142 (8, 2,920)	593 (93)	236 (2, 3,840)	3,490 (91)
NTproBNP	1,960 (11, 1,75,000)	2,466 (92)	2,010 (74, 1,75,000)	254 (88)	2,200 (11, 1,75,000)	2,634 (92)	2,020 (11, 1,75,000)	1,376 (90)	2,520 (123, 17,600)	596 (93)	2,080 (11, 1,75,000)	3,531 (92)
A1c	6 (4, 19)	1,029 (39)	6 (5, 15)	107 (37)	6 (4, 19)	1,091 (38)	6 (4, 18)	529 (35)	6 (4, 14)	320 (50)	6 (4, 19)	1,556 (41)
Medications												
Unique medications	22 (1, 134)	34 (1)	21 (1, 104)	7 (2)	22 (1, 134)	32 (1)	24 (1, 127)	16 (1)	9 (1, 105)	250 (39)	20 (1, 134)	291 (8)
Loop diuretic	1,707 (64)		198 (68)		1,883 (65)		1,111 (73)		130 (20)		2,157 (56)	
Thiazide diuretic	375 (14)		34 (11)		389 (13)		229 (15)		31 (4)		478 (12)	
SGLT2i	49 (1)		11 (3)		49 (1)		34 (2)		2 (0)		56 (1)	
Non-EBM	870 (32)		81 (28)		865 (30)		467 (30)		77 (12)		1,076 (28)	
EBMBB	2,249 (84)		262 (90)		2,644 (92)		1,348 (88)		100 (15)		2,787 (72)	
MRA	1,213 (45)		220 (76)		1,281 (44)		1,329 (87)		36 (5)		1,427 (37)	
ACEi/ARB	2,482 (93)		264 (91)		2,291 (80)		1,287 (84)		106 (16)		2,672 (69)	
ARNI	175 (6)		170 (58)		167 (5)		137 (9)		6 (0)		183 (4)	
Ivabradine	26 (1)		9 (3)		25 (0)		21 (1)				26 (0)	
Digoxin	3 (0)				3 (0)		1 (0)				3 (0)	
Comorbidities												
Diabetes	1,332 (49)		153 (52)		1,428 (50)		791 (52)		248 (38)		1,856 (48)	
Atrial fibrillation	1,159 (43)		132 (45)		1,296 (45)		688 (45)		240 (37)		1,698 (44)	
Coronary artery disease	2,050 (76)		206 (71)		2,229 (78)		1,185 (77)		401 (62)		2,862 (74)	
Nonischemic cardiac	618 (23)		83 (28)		628 (22)		337 (22)		238 (37)		970 (25)	
Hypertension	2,493 (93)		269 (93)		2,666 (93)		1,446 (95)		503 (78)		3,478 (90)	
Asthma	968 (36)		108 (37)		1,058 (37)		578 (38)		197 (30)		1,373 (35)	
COPD	579 (21)		47 (16)		638 (22)		332 (21)		121 (18)		830 (21)	
Depression	338 (12)		43 (14)		373 (13)		203 (13)		73 (11)		486 (12)	
Tobacco	753 (28)		65 (22)		782 (27)		432 (28)		181 (28)		1,047 (27)	
Procedures												
CRT	39 (1)		5 (1)		43 (1)		35 (2)		5 (0)		53 (1)	
ICD	420 (15)		71 (24)		468 (16)		305 (20)		54 (8)		553 (14)	
VAD	56 (2)		6 (2)		49 (1)		44 (2)		4 (0)		73 (1)	
RHC	131 (4)		36 (12)		125 (4)		111 (7)		28 (4)		183 (4)	

ICD, implantable cardioverter defibrillator; ACE/ARB, angiotensin converting enzyme inhibitor/angiotensin receptor blocker; ARNI, angiotensin receptor-neprilysin inhibitor; BB, evidence-based beta blocker; MRA, mineralocorticoid receptor antagonists; CRT, cardiac resynchronization therapy; VAD, ventricular assist device; RHC, right heart catheterization; EBM, evidence based medicine; SBP, systolic blood pressure; DBP, diastolic blood pressure; BUN, blood urea nitrogen; eGFR, estimated glomerular filtration rate; BNP, B-type natriuretic protein; NTproBNP, N-terminal pro-B-type natriuretic peptide; EBMBB, evidence-based medicine beta blocker; ACEi, angiotensin-converting enzyme inhibitor; COPD, chronic obstructive pulmonary disease.

### 3.2. Model performance

Table 2 presents the performance metrics of the different models for each medication type.

**Table 2:** Performance metrics of the different models for each medication outcome of interest.

Outcome	Modeling technique	AUC (95% CI)	Brier Score (95% CI)
ACE/ARB	Decision tree	0.744 (0.714–0.748)	0.171 (0.170–0.173)
	Random forest	**0.791** **(0.788–0.794)**	**0.158** **(0.157–0.159)**
	Logistic regression: lasso	0.783 (0.780–0.786)	0.162 (0.161–0.163)
	Logistic regression: ridge	0.780 (0.777–0.783)	0.163 (0.162–0.164)
	Logistic regression: elastic net	0.782 (0.779–0.785)	0.162 (0.161–0.163)
ARNI	Decision tree	0.788 (0.783–0.792)	0.146 (0.144–0.148)
	Random forest	**0.821** **(0.816–0.826)**	**0.063** **(0.063–0.064)**
	Logistic regression: lasso	0.820 (0.815–0.824)	0.155 (0.154–0.157)
	Logistic regression: ridge	0.816 (0.811–0.821)	0.156 (0.154–0.157)
	Logistic regression: elastic net	0.818 (0.813–0.823)	0.155 (0.153–0.156)
BB	Decision tree	0.753 (0.749–0.756)	0.150 (0.149–0.151)
	Random forest	**0.797** **(0.794–0.800)**	**0.140** **(0.139–0.141)**
	Logistic regression: lasso	0.789 (0.786–0.793)	0.143 (0.142–0.144)
	Logistic regression: ridge	0.789 (0.786–0.793)	0.143 (0.142–0.144)
	Logistic regression: elastic net	0.790 (0.786–0.793)	0.143 (0.142–0.144)
MRA	Decision tree	0.717 (0.713–0.720)	0.207 (0.206–0.209)
	Random forest	**0.788** **(0.786–0.791)**	**0.185** **(0.184–0.186)**
	Logistic regression: lasso	0.786 (0.783–0.789)	0.184 (0.182–0.185)
	Logistic regression: ridge	0.784 (0.781–0.787)	0.184 (0.183–0.185)
	Logistic regression: elastic net	0.785 (0.782–0.788)	0.184 (0.183–0.185)

AUC, area under the receiver operating characteristic curve; ACE/ARB, angiotensin converting enzyme inhibitor/angiotensin receptor blocker; ARNI, angiotensin receptor-neprilysin inhibitor; BB, evidence-based beta blocker; MRA, mineralocorticoid receptor antagonists.

Performance metrics for the random forest models are in bold.

The random forest was the best-performing model across all medication categories: ACE/ARB (AUC = 0.791, Brier score = 0.158), ARNI (AUC = 0.821, Brier score = 0.063), evidence-based BB (AUC = 0.797, Brier score = 0.140), and MRA (AUC = 0.788, Brier score = 0.185). For each of the medication categories, by Delong's test, there were not statistically significant differences at the 0.05 level between the AUCs for the random forest, elastic net, lasso, and ridge regression methods, although the best-performing models (random forests) consistently had significantly higher AUCs than the decision trees.

### 3.3. Top features for predicting prescribing

**Figure 2:**
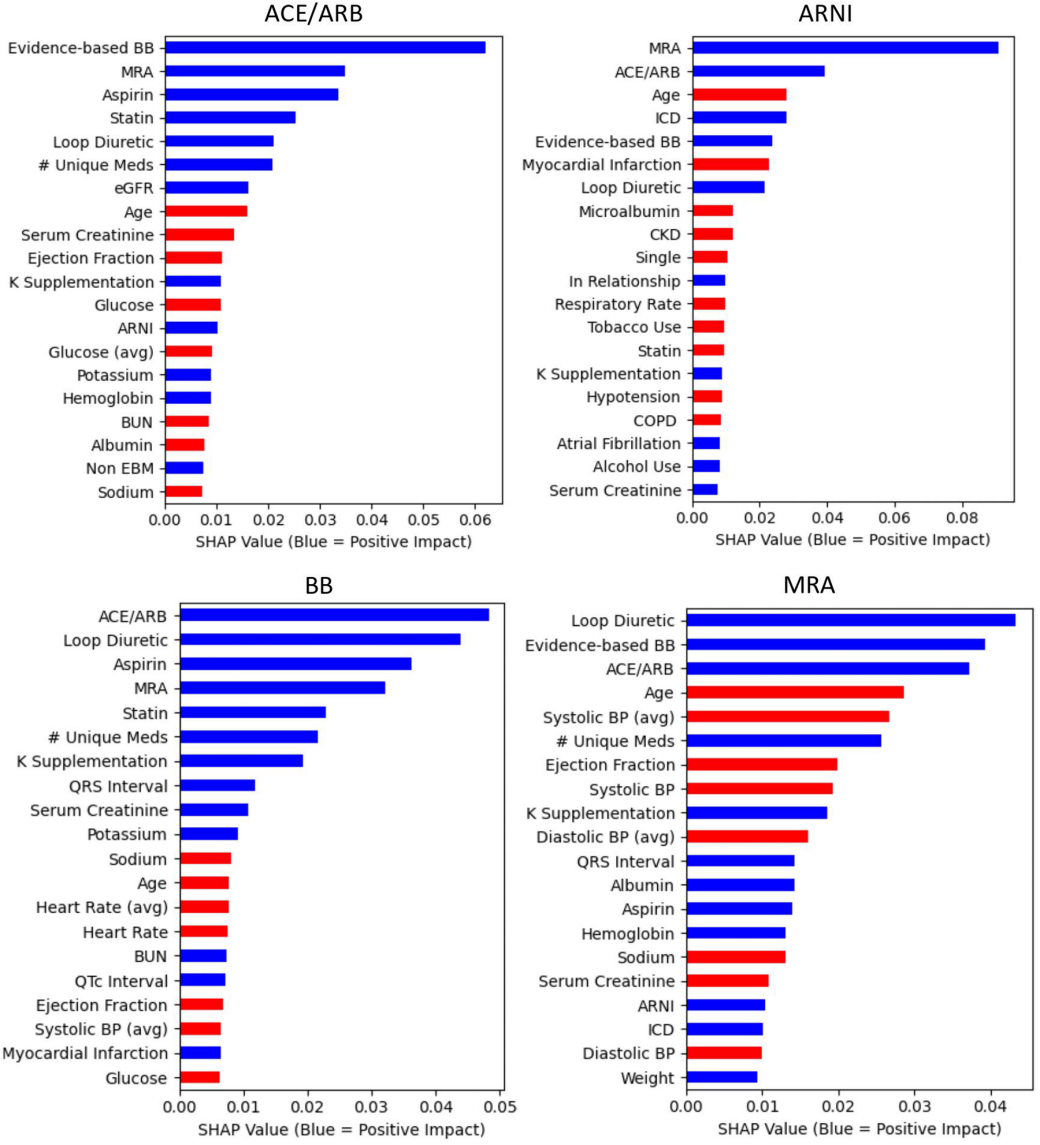
Central illustration. Top 20 predictor variables from the random forests by medication outcome Random forest feature importance is defined by SHAP values. Mean values are presented based on 100 cross-validation sampling estimates. With the exception of variables with “avg,” all variables are the values most proximal (most recent) to the Index date of the medication outcome being prescribed. “Avg” indicates these variables represent the mean of the two most recent values (most proximal to the index date). The albumin predictor variable is a binary indicator of availability of an albumin value at the time of prescription. For continuous variables, red indicates lower values predict the outcome (e.g., lower heart rates predict 88 prescribing). For categorical variables, red indicates that absence predicts the outcome (e.g., absence of a CKD diagnosis predicts ARNI prescribing). ARNI, angiotensin receptor-neprilysin inhibitor.

Figure 2 illustrates the top 20 features of importance (limited for visual clarity) for each medication outcome sorted by the mean absolute SHAP value. See Supplementary Appendix S2 for all features of importance for each of the medication outcomes.

### 3.4. Top predictors by medication outcome

Among the top 20 predictors for ACE/ARB prescribing were higher values of potassium and estimated glomerular filtration rate (eGFR) and lower values of serum creatinine and BUN. For prescription of ARNI, some of the strongest predictors were younger age and implantable cardioverter defibrillator (ICD). Other predictors among the top 20 for ARNI included being in a relationship (not single), absence of tobacco use, and no diagnosis of chronic kidney disease (CKD), chronic obstructive pulmonary disease (COPD), or hypotension. Among the top 20 predictors for evidence-based BB prescribing were lower values of heart rate, systolic blood pressure, and glucose values. Among the strongest predictors for MRA prescribing were younger age and lower systolic blood pressure values. Other predictors among the top 20 for MRA included lower values of diastolic blood pressure and serum creatinine.

### 3.5. Top predictors across all medication outcomes

Across all medication outcomes, prescribing of at least two of the other three medication types of interest was consistent among the top five predictors. All three other medications of interest (ACE/ARB, evidence-based BB, and MRA) were among the top five predictors for prescribing ARNI. Prescription of an ARNI was not among the top 10 predictors of prescribing an ACE/ARB or MRA, nor among the top 20 predictors of prescribing an evidence-based BB. For all medication outcomes, prescription of a loop diuretic and a statin were among the top 20 predictors. With the exception of evidence-based BB, younger age was among the top 10 predictors for all medication outcomes. Greater number of medications and lower ejection fraction values were also among the top 20 predictors for all medication outcomes, except ARNI.

## 4. Discussion

We applied ML methods to data readily available within most EHRs to identify predictors of prescribing evidence-based medications for HFrEF at our health system. We found that several ML methods performed similarly well for predicting prescribing of guideline-directed medical therapy (GDMT). From the random forest models, we identified multiple predictors of prescribing ACE/ARB, ARNI, evidence-based BB, and MRAs that can be used to guide the development of targeted strategies to improve prescribing for HFrEF and inform hypotheses for future study. Our application of machine learning approaches and the use of SHAP to identify predictors of prescribing are unique and strengths of this study. By exploring the use of ML algorithms to build predictive models for prescribing, we take advantage of their ability to account for possible interactions, collinearity, and nonlinear effects that commonly exist in complex healthcare data (40–43). By using SHAP, a model-agnostic approach to identify feature importance, we were able to quantify the directionality and importance of the relationship between the predictors and medication outcomes. SHAP additionally has an implementation to explain the modeling of local interaction and nonlinear predictor effects (31, 32), which can be explored in future studies to further investigate predictor behavior*.* Another strength of our study is the use of real-world EHR data, in contrast to data sources that are narrowly focused on a specific issue or setting or that rely on data generated from tightly controlled clinical trials (43, 33). We also included diverse social history data available within EHRs (e.g., substance use, primary language, relationship status), which facilitates the evaluation of more holistic factors influencing prescribing decisions. The use of EHR data allowed for evaluation of a large set of routinely collected data to detect predictor variables. In this setting, we found that a penalized logistic regression performed similarly to the best-performing ML approach; however, our approach for model-building and model-agnostic explanation allows for the evaluation of ML algorithms that may have superior predictive performance when applied to future explorations of complex EHR data. This model-building approach to defining predictors of prescribing is actively being used by our health system to strategically design interventions to overcome barriers to evidence-based prescribing. As the context and evidence change, this approach to using ML to define predictors of prescribing can be repeated to identify new issues and design corresponding solutions for them.

Some predictors of prescribing that we identified align with findings from past studies, which serves to triangulate our findings. For example, worse renal function is a well-known barrier to prescribing ACE/ARB, ARNI, and MRA (34–36). In our study, lower values of serum creatinine, which suggests better renal function, was among the top 20 predictors of prescribing for ACE/ARB and MRA. Although higher values of serum creatinine was among the top 20 predictors for ARNI, the absence of a CKD diagnosis was also one. Patients prescribed an ARNI may have higher serum creatinine values within the range of normal renal function. The finding of better renal function as a predictor of prescribing confirms the presence of this modifiable knowledge issue, for which our health system is actively designing interventions, such as tailored clinical decision support (CDS) tools and education to address.

Past studies also point to hyperkalemia or concerns of precipitating hyperkalemia as common barriers to prescribing ACE/ARB, ARNI, and MRA (37). Interestingly, in our study, the absence of a hyperkalemia diagnosis was not among the top 20 predictors for prescribing these medications, but potassium supplementation was for ACE/ARB, ARNI, and MRA. Furthermore, higher values of serum potassium was a top predictor for ACE/ARB. These discrepancies between our findings and past studies may suggest that concerns related to hyperkalemia are not as influential of a predictor after accounting for the complex interacting effects of other key predictors. Additional studies are needed to evaluate the generalizability of this finding in other health systems. Based on these findings, our health system is not currently prioritizing resources to design interventions to address any modifiable informational needs related to hyperkalemia.

Another common barrier to prescribing these medications are concerns related to hypotension across all medications and bradycardia for evidence-based BBs (38–40). This aligns well with our findings of lower blood pressures and heart rates being among the top predictors for prescribing evidence-based BBs (heart rate and blood pressure) and MRAs (blood pressure only). However, blood pressure was not among the top predictors of prescribing an ACE/ARB or ARNI. The absence of blood pressure as a top predictor for ACE/ARB or ARNI may suggest that clinicians are less concerned about hypotension with these specific medications, but requires further investigation to understand these findings within our health system and externally. In the case of ACE/ARBs specifically, there is a large therapeutic dose range and it is possible that the absence of blood pressure as a predictor is because lower doses are prescribed to these patients to mitigate clinician concerns.

There were also some notable findings when comparing predictors of prescribing ARNI to the other medication outcomes. We found that patients prescribed an ARNI were more likely to be in a relationship (not single) and be nontobacco users, which may suggest a stronger support system and choice of healthier lifestyle choices. A study conducted in Sweden also found that patients in a relationship were more likely to be prescribed ARNI (41). In our study, the absence of CKD, COPD, and hypotension were among the top predictors, while greater number of medications was not among the top predictors (it was the 70th most important predictor; see Supplementary Appendix B), which further supports the notion that these patients were healthier. Younger age was also the third strongest predictor of ARNI prescription. Other studies have also associated younger age (41–44) and fewer comorbidities (43) with ARNI prescription. Although we found the absence of a CKD diagnosis was a predictor of ARNI, other studies report conflicting findings regarding the association with CKD (42–44), which may be related to differences in the types (inpatient, outpatient, EHR, claims), volume, or variety of data used. At our institution, we are continuing to explore these findings and any potential solutions by conducting qualitative interviews.

The higher relative cost of ARNI (45) may, in part, explain some of the predictors uniquely associated with ARNI prescription in our study. Younger patients with greater support systems who have historically made healthier lifestyle choices (tobacco abstinence) may be more likely to choose to invest in more expensive medications. It is also possible that there is implicit or explicit clinician bias that results in clinicians being more likely to selectively recommend an ARNI to this demographic. Others have found that patients of higher socioeconomic status are more likely to be prescribed an ARNI (42). In our study, we did not find commercial insurance, race, or ethnicity predictive of ARNI, but others have found that non-Hispanic patients are more likely to be prescribed an ARNI (42).

Similar to other studies, patients prescribed an ARNI were more likely to be prescribed other guideline-concordant therapies (42–44); the three other evidence-based medications and an ICD were some of the strongest predictors. Although not a new finding (42–44), the fact that we were able to reproduce this using ML, which more rigorously considers the complex interactions of diverse variables, supports the validity of our other findings. ARNIs are more likely to be prescribed by cardiologists who are more focused on optimizing management of HFrEF and who may be more familiar with the evidence and comfortable applying it than other providers (42, 46).

This study does have several limitations. EHR data often do not include some important variables, such as social determinants of health or patient-reported outcomes, which are known to influence patient care (42, 47). Our study also does not allow for evaluation or interpretation of temporal relationships between our predictor variables and medication outcomes. For example, we were limited to dates of prescriptions and unable to identify which medications a patient was taking or actively prescribed at a specific point in time, which is an inherent limitation of EHR data in nonintegrated health systems. As such, ACE/ARB as a predictor of prescribing ARNI should be interpreted as prescription of an ACE/ARB in the past year is associated with prescribing ARNI. Furthermore, given the inherent limitations in accurately measuring medication doses with EHR data, we did not evaluate doses of target medications or duration of medication use, but these are important areas for future inquiry. We also did not exclude absolute contraindications to the medication outcomes of interest; however, such contraindications are infrequent, and thus, this is unlikely to have significant impact our findings.

Although our model has not been validated in an external data set, this approach is highly relevant for health systems seeking to improve prescribing for this high risk population. Using this approach in our health system, we identified salient predictors of suboptimal prescribing that will be used to strategically design tailored interventions to address barriers to prescribing evidence-based medications for HFrEF, notably educational initiatives and CDS embedded within the EHR. For some predictors, such as a patient's relationship status, qualitative evaluations are needed to shed light on why these predictors influence prescribing before tailored interventions can be implemented. Although not all predictors can be tangibly addressed (relationship status), many are modifiable (e.g., clinician misconceptions or knowledge gaps). It is possible that through additional qualitative exploration, we identify that certain predictors such as relationship status have underlying drivers that are modifiable. Future research could also explore subgroup analyses for certain patient groups to better understand why they were not prescribed evidence-based medications. Given the persistence of nonadherence to evidence-based prescribing over time despite many concerted efforts to close the gaps, reimagined and multimodal interventions may be required to significantly move the needle. This study can assist in identifying modifiable barriers (e.g., salient knowledge gaps or misconceptions) to design targeted interventions for HFrEF. Types of interventions may include traditional modes of education to address knowledge gaps, direct to patient activation strategies (18), or integration of emerging technologies and artificial intelligence to translate evidence-based recommendations into clinician workflows at the right time (48). For example, perhaps patient-facing CDS tools delivered via EHR patient portals in combination with clinician-facing CDS tools and audit and feedback with peer-benchmarking are needed to improve the representativeness of prescribing ARNIs to patients of diverse backgrounds. In this example, CDS tools empower patients and remind or guide clinicians, while clinicians are also held accountable through social norming.

## Data Availability

The data analyzed in this study is subject to the following licenses/restrictions: Any data shared will be deidentified. Requests to access these datasets should be directed to the corresponding author.
