# Supplementary material for: A machine learning evaluation of patient characteristics associated with prescribing of guideline-directed medical therapy for heart failure
Source: Front Cardiovasc Med. 2023 Jun 21;10:1169574. doi: 10.3389/fcvm.2023.1169574 (PMC10321403; doi:10.3389/fcvm.2023.1169574)
Supplement: Supplementary file 1 [file Datasheet1.docx]

| **Appendix 1.** Model input variable definitions | Page 2 |
| --- | --- |
| **Appendix 2.** Random forest features of interest for each medication outcome of interest (Gini index and 95% Confidence Intervals) | Page 9 |

**Appendix 1. Model input variable definitions**

| **Model input variable** | **Definition** |
| --- | --- |
| Hispanic ethnicity | Patient reported ethnicity |
| Non-Hispanic ethnicity | Patient reported ethnicity |
| Other ethnicity | Patient reported ethnicity |
| Insurance type: commercial | Most recent insurance status documented in EHR |
| Insurance type: Medicaid/Dual | Most recent insurance status documented in EHR |
| Insurance type: Medicare/Tricare | Most recent insurance status documented in EHR |
| Insurance type: Other | Most recent insurance status documented in EHR |
| Insurance type: Self pay | Most recent insurance status documented in EHR |
| BNP >250 ng/L | Most recent value |
| BNP ≤ 250 ng/L | Most recent value |
| BNP missing | Most recent value |
| NTproBNP ≥ 1000 ng/L | Most recent value |
| NTproBNP < 1000 ng/L | Most recent value |
| NTproBNP missing | Most recent value |
| Calcium 8.4-10.6 mg/dL | Most recent value |
| Calcium > 10.6 mg/dL | Most recent value |
| Calcium < 8.4 mg/dL | Most recent value |
| Calcium missing | Most recent value |
| Microalbumin ≥ 30 mg/dL | Most recent value |
| Microalbumin < 30 mg/dL | Most recent value |
| Microalbumin missing | Most recent value |
| Hemoglobin A1C 6.5-7.5% | Most recent value |
| Hemoglobin A1C ≥ 7.5% | Most recent value |
| Hemoglobin A1C < 6.5% | Most recent value |
| Hemoglobin A1C missing | Most recent value |
| Black race | Patient reported race |
| Other race | Patient reported race as a different category than listed or as a combination of categories |
| Unknown race | Patient reported race unknown |
| White race | Patient reported race |
| Relationship status: In relationship | Patient reported relationship status |
| Relationship status: Single | Patient reported relationship status |
| Relationship status: Other | Patient reported relationship status as a different category than ‘single’ or ‘in relationship’ |
| Language: English | Patient reported preferred language |
| Language: Other/Unknown | Patient reported preferred language |
| Language: Spanish | Patient reported preferred language |
| Ventricular assist device | Procedure recorded sometime in the past using Colorado's state-wise all-payers claims database |
| Cardiac resynchronization therapy | Procedure recorded sometime in the past using Colorado's state-wise all-payers claims database |
| ICD | Procedure recorded sometime in the past using Colorado's state-wise all-payers claims database |
| Right heart catheterization | Procedure recorded sometime in the past using Colorado's state-wise all-payers claims database |
| Ivabradine | Prescribed in past year |
| Metformin | Prescribed in past year |
| Sulfonylurea | Prescribed in past year |
| Thiazolidinedione | Prescribed in past year |
| Insulin | Prescribed in past year |
| Inhaled anticholinergic | Prescribed in past year |
| Glucagon-like peptide-1 receptor agonist | Prescribed in past year |
| Alpha agonist | Prescribed in past year |
| Amlodipine | Prescribed in past year |
| Nifedipine | Prescribed in past year |
| Non-dihydropyridine calcium channel blocker | Prescribed in past year |
| K supplement | Prescribed in past year |
| Patiromer | Prescribed in past year |
| Sodium zirconium | Prescribed in past year |
| Sodium polystyrene | Prescribed in past year |
| Statin | Prescribed in past year |
| Ezetimibe | Prescribed in past year |
| PCSK9 inhibitor | Prescribed in past year |
| Aspirin | Prescribed in past year |
| Clopidogrel | Prescribed in past year |
| Prasugrel | Prescribed in past year |
| Ticagrelor | Prescribed in past year |
| DOAC | Prescribed in past year |
| Cilostazol | Prescribed in past year |
| Clonidine | Prescribed in past year |
| Non-EBM BB | Prescribed in past year |
| Metoprolol tartrate | Prescribed in past year |
| Evidence-based BB | Prescribed in past year |
| MRA | Prescribed in past year |
| ARNI | Prescribed in past year |
| ACE/ARB | Prescribed in past year |
| Digoxin | Prescribed in past year |
| Sotalol | Prescribed in past year |
| Aliskiren | Prescribed in past year |
| Warfarin | Prescribed in past year |
| SGLT2 inhibitor | Prescribed in past year |
| Thiazide | Prescribed in past year |
| Loop diuretic | Prescribed in past year |
| Diabetes | Diagnosis documented sometime in the past based on ICD codes |
| CKD | Diagnosis documented sometime in the past based on ICD codes |
| COPD | Diagnosis documented sometime in the past based on ICD codes |
| COPD phenotype | Diagnosis documented sometime in the past based on presence of COPD ICD codes and absence of certain ICD codes such as those for asthma |
| COPD | Diagnosis documented sometime in the past based on ICD codes |
| Atrial fibrillation | Diagnosis documented sometime in the past |
| Asthma phenotype | Diagnosis documented sometime in the past based on presence of asthma ICD codes and absence of certain ICD codes such as those for COPD |
| Asthma | Diagnosis documented sometime in the past based on ICD codes |
| Coronary artery disease | Diagnosis documented sometime in the past based on ICD codes |
| Nonischemic cardiomyopathy | Diagnosis documented sometime in the past based on ICD codes |
| Myocardial infarction | Diagnosis documented sometime in the past based on ICD codes |
| Depression diagnosis | Diagnosis documented sometime in the past based on ICD codes |
| Anxiety diagnosis | Diagnosis documented sometime in the past based on ICD codes |
| Hypertension | Diagnosis documented sometime in the past based on ICD codes |
| Hyperkalemia | Diagnosis documented sometime in the past based on ICD codes |
| Hypotension | Diagnosis documented sometime in the past based on ICD codes |
| Angioedema | Diagnosis documented sometime in the past based on ICD codes |
| ACE-induced angioedema | Diagnosis documented sometime in the past based on ICD codes considering temporal relationship with ACE prescription |
| Height | Most recent value |
| Systolic BP | Most recent value |
| Systolic BP (avg) | Mean of two most recent values |
| Diastolic BP | Most recent value |
| Diastolic BP (avg) | Mean of two most recent values |
| Heart rate | Most recent value |
| Heart rate (avg) | Mean of two most recent values |
| Weight | Most recent value |
| Respiratory rate | Most recent value |
| Serum creatinine | Most recent value |
| International normalized ratio | Most recent value |
| Bilirubin | Most recent value |
| Prothrombin time | Most recent value |
| Albumin | Most recent value |
| Hemoglobin | Most recent value |
| Glucose | Most recent value |
| Glucose (avg) | Mean of two most recent values |
| Sodium | Most recent value |
| eGFR | Most recent value |
| Potassium | Most recent value |
| ALT | Most recent value |
| QRS interval | Most recent value |
| QTc interval | Most recent value |
| Ejection fraction | Most recent value |
| BUN | Most recent value |
| # Unique medications | Number of unique medications based on 10-digit Generic Product Identifier (GPI) codes in the past year |
| BMI | Most recent value |
| Age | As of the index date |
| Gender | Patient reported gender |
| Social security number | Binary indicator of presence of a documented value |
| Tobacco use | Patient reported use |
| Alcohol use | Patient reported use |
| Interpreter needed | Binary indicator of interpreter needed |
| * All candidate predictor variables were collected on or before the index date | |

**Appendix 2. Random forest features of interest for each medication outcome of interest (SHAP values and correlation coefficients)**

| **Random forest features of importance for beta blocker prescribing** | **SHAP value** | **Correlation Coefficient** |
| --- | --- | --- |
| ACE/ARB | 0.04832 | 0.88054 |
| Loop Diuretic | 0.04388 | 0.87498 |
| Aspirin | 0.03631 | 0.89065 |
| MRA | 0.03223 | 0.86623 |
| Statin | 0.02283 | 0.77881 |
| # Unique Medications | 0.02169 | 0.62515 |
| K supplement | 0.01936 | 0.81366 |
| QRS Interval | 0.01190 | 0.54875 |
| Serum Creatinine | 0.01086 | 0.14219 |
| Potassium | 0.00911 | 0.51328 |
| Sodium | 0.00805 | -0.39440 |
| Age | 0.00777 | -0.40753 |
| Heart Rate (avg) | 0.00771 | -0.44304 |
| Heart Rate | 0.00755 | -0.41325 |
| BUN | 0.00742 | 0.17786 |
| QTc Interval | 0.00714 | 0.23659 |
| Ejection Fraction | 0.00686 | -0.16611 |
| Systolic BP (avg) | 0.00654 | -0.17485 |
| Myocardial Infarction | 0.00653 | 0.63132 |
| Glucose | 0.00630 | -0.12449 |
| Nonischemic Cardiomyopathy | 0.00624 | -0.68558 |
| Systolic BP | 0.00614 | -0.17224 |
| Glucose (avg) | 0.00600 | -0.29796 |
| Inhaled anticholinergic | 0.00596 | 0.72217 |
| ICD | 0.00584 | 0.83631 |
| Hemoglobin | 0.00545 | 0.17864 |
| Prothrombin time | 0.00544 | -0.21398 |
| ALT | 0.00539 | -0.26979 |
| Diastolic BP (avg) | 0.00533 | -0.24192 |
| Coronary artery disease | 0.00525 | 0.69314 |
| BMI | 0.00520 | 0.08366 |
| Insulin | 0.00515 | 0.66381 |
| Non-EBM BB | 0.00511 | -0.66420 |
| Weight | 0.00502 | 0.05279 |
| Diastolic BP | 0.00489 | -0.22074 |
| eGFR | 0.00489 | -0.29168 |
| International normalized ratio | 0.00487 | -0.22187 |
| Height | 0.00457 | -0.09457 |
| Albumin | 0.00448 | -0.34193 |
| Non-dihydropyridine calcium channel blocker | 0.00424 | 0.74247 |
| Bilirubin | 0.00411 | -0.07949 |
| Hypertension | 0.00392 | 0.76482 |
| Respiratory Rate | 0.00376 | -0.52176 |
| Hemoglobin A1C missing | 0.00343 | -0.72278 |
| Warfarin | 0.00287 | -0.06394 |
| DOAC | 0.00260 | 0.61931 |
| Metoprolol tartrate | 0.00232 | -0.66226 |
| Ventricular assist device | 0.00219 | -0.81533 |
| Clopidogrel | 0.00203 | 0.41533 |
| Relationship status: Other | 0.00200 | -0.75820 |
| Asthma | 0.00194 | 0.40714 |
| Hemoglobin A1C < 6.5% | 0.00178 | 0.64009 |
| COPD | 0.00172 | 0.66383 |
| Right heart catheterization | 0.00158 | -0.77219 |
| Metformin | 0.00151 | 0.38041 |
| Insurance type: Other | 0.00148 | -0.81156 |
| Insurance type: Medicare/Tricare | 0.00143 | 0.45459 |
| Hypotension | 0.00120 | 0.38142 |
| Sotalol | 0.00119 | -0.81312 |
| Non-Hispanic ethnicity | 0.00112 | 0.75952 |
| Alpha agonist | 0.00110 | -0.61034 |
| Hyperkalemia | 0.00109 | 0.14489 |
| Relationship status: Single | 0.00107 | 0.33687 |
| Atrial Fibrillation | 0.00104 | -0.11333 |
| NTproBNP missing | 0.00102 | 0.59312 |
| Diabetes | 0.00101 | -0.15834 |
| Tobacco Use | 0.00100 | -0.41299 |
| Amlodipine | 0.00099 | -0.54022 |
| Relationship status: In a relationship | 0.00099 | 0.05180 |
| Thiazide | 0.00095 | -0.37724 |
| Hispanic ethnicity | 0.00094 | -0.77260 |
| NTproBNP ≥ 1000 ng/L | 0.00088 | -0.65284 |
| Hemoglobin A1C 6.5-7.5% | 0.00087 | -0.72895 |
| Insurance type: commercial | 0.00087 | -0.66919 |
| Alcohol Use | 0.00087 | 0.34585 |
| COPD | 0.00084 | -0.33922 |
| Social security number | 0.00082 | -0.71492 |
| CKD | 0.00082 | 0.23890 |
| Calcium < 8.4 mg/dL | 0.00079 | -0.62438 |
| Microalbumin ≥ 30 mg/dL | 0.00075 | -0.50311 |
| Female | 0.00074 | -0.02365 |
| COPD phenotype | 0.00072 | -0.82833 |
| Language: Spanish | 0.00071 | -0.74832 |
| White race | 0.00065 | -0.16441 |
| Calcium missing | 0.00064 | 0.47239 |
| Other race | 0.00061 | -0.59936 |
| Insurance type: Medicaid/Dual | 0.00060 | 0.35039 |
| Anxiety diagnosis | 0.00059 | -0.75586 |
| NTproBNP < 1000 ng/L | 0.00058 | -0.78420 |
| Microalbumin missing | 0.00058 | -0.20275 |
| Hemoglobin A1C ≥ 7.5% | 0.00051 | -0.00104 |
| Asthma phenotype | 0.00048 | -0.67083 |
| ARNI | 0.00047 | 0.38382 |
| BNP missing | 0.00046 | -0.19662 |
| Depression diagnosis | 0.00046 | -0.32031 |
| Glucagon-like peptide-1 receptor agonist | 0.00046 | -0.64668 |
| Clonidine | 0.00040 | -0.64606 |
| Sulfonylurea | 0.00039 | 0.38113 |
| Insurance type: Self pay | 0.00037 | -0.55981 |
| Language: English | 0.00035 | 0.69456 |
| Unknown race | 0.00035 | -0.75852 |
| Black race | 0.00035 | 0.18105 |
| SGLT2 inhibitor | 0.00034 | -0.69594 |
| Angioedema | 0.00031 | -0.64672 |
| Microalbumin < 30 mg/dL | 0.00029 | -0.11340 |
| BNP >250 ng/L | 0.00028 | -0.07233 |
| Other ethnicity | 0.00025 | -0.60323 |
| BNP ≤250 ng/L | 0.00023 | -0.18101 |
| Ezetimibe | 0.00023 | -0.05192 |
| ACE-induced angioedema | 0.00021 | -0.72419 |
| Interpreter | 0.00021 | -0.49593 |
| Ticagrelor | 0.00016 | -0.46959 |
| Prasugrel | 0.00013 | -0.67932 |
| Cardiac resynchronization therapy | 0.00011 | -0.68025 |
| Sodium polystyrene | 0.00009 | -0.27374 |
| Language: Other/Unknown | 0.00007 | -0.51519 |
| Nifedipine | 0.00006 | -0.42979 |
| Cilostazol | 0.00005 | -0.56302 |
| PCSK9 inhibitor | 0.00004 | 0.79439 |
| Ivabradine | 0.00004 | 0.20584 |
| Calcium 8.4-10.6 mg/dL | 0.00003 | -0.50735 |
| Thiazolidinedione | 0.00002 | -0.05933 |
| Patiromer | 0.00001 | -0.56947 |
| Calcium > 10.6 mg/dL | 0.00001 | -0.59321 |
| Sodium zirconium | 0.00000 | 0.64993 |
| Digoxin | 0.00000 | 0.20422 |
| Aliskiren | 0.00000 | 0.00000 |

| **Random forest features of importance for mineralocorticoid receptor antagonist prescribing** | **SHAP Value** | **Correlation Coefficient** |
| --- | --- | --- |
| Loop Diuretic | 0.04335 | 0.90129 |
| Evidence-based BB | 0.03922 | 0.92993 |
| ACE/ARB | 0.03722 | 0.91592 |
| Age | 0.02855 | -0.74579 |
| Systolic BP (avg) | 0.02665 | -0.72421 |
| # Unique medications | 0.02566 | 0.68782 |
| Ejection Fraction | 0.01985 | -0.52292 |
| Systolic BP | 0.01923 | -0.68746 |
| K supplement | 0.01857 | 0.77319 |
| Diastolic BP (avg) | 0.01596 | -0.55590 |
| QRS Interval | 0.01427 | 0.70879 |
| Albumin | 0.01422 | 0.02167 |
| Aspirin | 0.01398 | 0.78502 |
| Hemoglobin | 0.01313 | 0.62955 |
| Sodium | 0.01304 | -0.68806 |
| Serum Creatinine | 0.01090 | -0.43295 |
| ARNI | 0.01045 | 0.89335 |
| ICD | 0.01016 | 0.82418 |
| Diastolic BP | 0.00989 | -0.50651 |
| Weight | 0.00932 | 0.52421 |
| BMI | 0.00901 | 0.55152 |
| QTc Interval | 0.00851 | 0.38049 |
| Potassium | 0.00848 | 0.58704 |
| eGFR | 0.00750 | 0.71463 |
| BUN | 0.00738 | 0.47128 |
| Microalbumin missing | 0.00735 | -0.78068 |
| Bilirubin | 0.00732 | 0.49638 |
| Glucose (avg) | 0.00701 | -0.20737 |
| Prothrombin time | 0.00640 | -0.04794 |
| International normalized ratio | 0.00608 | -0.40274 |
| Heart Rate | 0.00606 | -0.02093 |
| Glucose | 0.00602 | 0.07854 |
| Heart Rate (avg) | 0.00600 | -0.16694 |
| ALT | 0.00600 | 0.09404 |
| Height | 0.00546 | 0.07765 |
| Hemoglobin A1C missing | 0.00545 | -0.66830 |
| Insurance type: Medicare/Tricare | 0.00505 | -0.64469 |
| Microalbumin < 30 mg/dL | 0.00429 | 0.84365 |
| Respiratory Rate | 0.00330 | 0.11254 |
| Metformin | 0.00294 | 0.76922 |
| White | 0.00293 | -0.71236 |
| Hypotension | 0.00288 | 0.72926 |
| Non-dihydropyridine calcium channel blocker | 0.00274 | 0.71408 |
| Statin | 0.00267 | 0.17838 |
| Hemoglobin A1C < 6.5% | 0.00229 | 0.68886 |
| Atrial Fibrillation | 0.00222 | 0.15345 |
| Diabetes | 0.00216 | 0.46897 |
| Right heart catheterization | 0.00209 | 0.81580 |
| Black race | 0.00197 | 0.73810 |
| CKD | 0.00192 | -0.63815 |
| DOAC | 0.00189 | 0.62118 |
| Insulin | 0.00183 | 0.52104 |
| Warfarin | 0.00157 | 0.52401 |
| Hypertension | 0.00152 | 0.73483 |
| Inhaled anticholinergic | 0.00142 | -0.16291 |
| Myocardial Infarction | 0.00139 | -0.00802 |
| Thiazide | 0.00138 | 0.65475 |
| Insurance type: Medicaid/Dual | 0.00137 | 0.69043 |
| NTproBNP missing | 0.00136 | -0.74231 |
| Relationship status: In a relationship | 0.00133 | 0.46204 |
| Asthma | 0.00129 | 0.41421 |
| Alcohol Use | 0.00124 | 0.49720 |
| Hyperkalemia | 0.00115 | -0.54142 |
| Tobacco Use | 0.00114 | -0.20465 |
| Alpha agonist | 0.00113 | -0.31971 |
| Non-EBM BB | 0.00110 | -0.04020 |
| Hemoglobin A1C 6.5-7.5% | 0.00107 | 0.68062 |
| Relationship status: Single | 0.00105 | -0.10952 |
| Coronary artery disease | 0.00103 | 0.30718 |
| Clopidogrel | 0.00103 | 0.52158 |
| Nonischemic cardiomyopathy | 0.00093 | -0.26002 |
| Calcium missing | 0.00091 | 0.27300 |
| Other race | 0.00090 | 0.65656 |
| Calcium < 8.4 mg/dL | 0.00090 | -0.20916 |
| Insurance type: commercial | 0.00088 | 0.45726 |
| Metoprolol | 0.00088 | 0.13509 |
| COPD | 0.00086 | -0.32393 |
| Female | 0.00084 | 0.15467 |
| NTproBNP ≥ 1000 ng/L | 0.00081 | 0.75753 |
| COPD | 0.00078 | -0.34689 |
| Depression diagnosis | 0.00075 | -0.43136 |
| Hispanic ethnicity | 0.00074 | 0.58925 |
| Relationship status: Other | 0.00069 | -0.71144 |
| Language: English | 0.00068 | -0.73743 |
| Amlodipine | 0.00066 | -0.14218 |
| Ivabradine | 0.00066 | 0.85789 |
| BNP missing | 0.00065 | -0.59565 |
| NTproBNP < 1000 ng/L | 0.00064 | 0.57710 |
| Microalbumin ≥ 30 mg/dL | 0.00063 | 0.42895 |
| Insurance type: Other | 0.00060 | 0.56095 |
| Hemoglobin A1C ≥ 7.5% | 0.00057 | 0.36181 |
| Non-Hispanic ethnicity | 0.00055 | -0.36101 |
| Interpreter needed | 0.00051 | 0.76361 |
| Sulfonylurea | 0.00051 | 0.21392 |
| Clonidine | 0.00049 | 0.70824 |
| Ticagrelor | 0.00049 | 0.72571 |
| Asthma phenotype | 0.00048 | 0.09496 |
| BNP >250 ng/L | 0.00044 | 0.62899 |
| Sotalol | 0.00044 | 0.67407 |
| Ventricular assist device | 0.00042 | 0.72221 |
| Language: Spanish | 0.00039 | 0.58458 |
| Ezetimibe | 0.00036 | 0.47709 |
| Cardiac resynchronization therapy | 0.00035 | 0.78252 |
| BNP ≤ 250 ng/L | 0.00032 | 0.44901 |
| Social security number | 0.00032 | -0.15174 |
| Sodium polystyrene | 0.00026 | -0.60160 |
| Anxiety diagnosis | 0.00023 | 0.58965 |
| Glucagon-like peptide-1 receptor agonist | 0.00021 | -0.07589 |
| COPD Phenotype | 0.00019 | 0.49341 |
| Other ethnicity | 0.00017 | -0.48906 |
| SGLT2 inhibitor | 0.00017 | 0.20976 |
| Nifedipine | 0.00013 | 0.83381 |
| Unknown race | 0.00013 | -0.41410 |
| Language: Other/Unknown | 0.00012 | 0.43936 |
| PCSK9 inhibitor | 0.00011 | 0.62383 |
| Angioedema | 0.00010 | -0.18351 |
| Cilostazol | 0.00010 | 0.65940 |
| Insurance type: Self pay | 0.00009 | -0.35615 |
| Thiazolidinedione | 0.00009 | -0.10019 |
| Prasugrel | 0.00006 | 0.52627 |
| ACE-induced angioedema | 0.00004 | -0.72680 |
| Calcium 8.4-10.6 mg/dL | 0.00004 | 0.57045 |
| Patiromer | 0.00001 | -0.71395 |
| Calcium > 10.6 mg/dL | 0.00000 | -0.66017 |
| Digoxin | 0.00000 | 0.00000 |
| Sodium zirconium | 0.00000 | 0.00000 |
| Aliskiren | 0.00000 | 0.00000 |

| **Random forest features of importance for angiotensin receptor-neprilysin inhibitor prescribing** | **SHAP Value** | **Correlation Coefficient** |
| --- | --- | --- |
| MRA | 0.09084 | 0.76084 |
| ACE/ARB | 0.03943 | 0.96897 |
| Age | 0.02806 | -0.57148 |
| ICD | 0.02806 | 0.31281 |
| Evidence-based BB | 0.02364 | 0.89891 |
| Myocardial Infarction | 0.02270 | -0.37859 |
| Loop Diuretic | 0.02139 | 0.43153 |
| Microalbumin missing | 0.01224 | -0.35481 |
| CKD | 0.01205 | -0.62712 |
| Relationship status: Single | 0.01050 | -0.18074 |
| Relationship status: In Relationship | 0.00993 | 0.34181 |
| Respiratory Rate | 0.00981 | -0.35957 |
| Tobacco Use | 0.00967 | -0.45797 |
| Statin | 0.00964 | -0.06424 |
| K supplement | 0.00897 | 0.16457 |
| Hypotension | 0.00884 | -0.26236 |
| COPD | 0.00858 | -0.54120 |
| Atrial Fibrillation | 0.00837 | 0.23768 |
| Alcohol Use | 0.00825 | 0.19421 |
| Serum Creatinine | 0.00770 | 0.19901 |
| Height | 0.00722 | 0.55727 |
| Asthma | 0.00715 | 0.13711 |
| Hemoglobin | 0.00709 | 0.57284 |
| Insulin | 0.00691 | -0.51661 |
| White race | 0.00678 | 0.41287 |
| Insurance type: Medicare/Tricare | 0.00622 | 0.22086 |
| Right heart catheterization | 0.00614 | 0.22883 |
| Aspirin | 0.00590 | 0.12435 |
| NTproBNP missing | 0.00562 | -0.24315 |
| Weight | 0.00537 | 0.46779 |
| Inhaled anticholinergic | 0.00533 | -0.55103 |
| Systolic BP (avg) | 0.00528 | -0.55041 |
| Ejection fraction | 0.00514 | -0.43905 |
| Gender | 0.00498 | -0.49699 |
| Warfarin | 0.00489 | -0.51288 |
| Systolic BP | 0.00471 | -0.53385 |
| Hemoglobin A1C < 6.5% | 0.00435 | -0.12580 |
| Sodium | 0.00417 | -0.41563 |
| Calcium missing | 0.00413 | 0.62620 |
| Potassium | 0.00408 | 0.41526 |
| Diabetes | 0.00402 | -0.02333 |
| Clopidogrel | 0.00397 | -0.34214 |
| Hemoglobin A1C missing | 0.00383 | 0.11762 |
| Non-EBM BB | 0.00378 | -0.43816 |
| Microalbumin < 30 mg/dL | 0.00369 | 0.22138 |
| Coronary artery disease | 0.00352 | 0.44390 |
| Metoprolol | 0.00352 | -0.53343 |
| Albumin | 0.00351 | 0.05839 |
| DOAC | 0.00348 | -0.02564 |
| International normalized ratio | 0.00326 | 0.31762 |
| Calcium < 8.4 mg/dL | 0.00324 | -0.58255 |
| Diastolic BP | 0.00322 | -0.31280 |
| Bilirubin | 0.00321 | -0.05720 |
| Insurance type: commercial | 0.00310 | 0.06158 |
| Insurance type: Medicaid/Dual | 0.00304 | 0.13267 |
| eGFR | 0.00295 | -0.04838 |
| BUN | 0.00293 | -0.03774 |
| Diastolic BP | 0.00290 | -0.22292 |
| Metformin | 0.00290 | 0.23504 |
| BMI | 0.00281 | 0.33833 |
| Nonischemic cardiomyopathy | 0.00276 | -0.22417 |
| Heart rate (avg) | 0.00270 | -0.33449 |
| QRS interval | 0.00265 | 0.32588 |
| Hyperkalemia | 0.00265 | -0.09986 |
| Amlodipine | 0.00258 | -0.74236 |
| Ticagrelor | 0.00244 | 0.23238 |
| ALT | 0.00238 | 0.01524 |
| Prothrombin time | 0.00230 | 0.26572 |
| Glucose | 0.00226 | 0.12920 |
| # Unique medications | 0.00218 | 0.14301 |
| Microalbumin ≥ 30 mg/dL | 0.00214 | 0.18422 |
| Heart rate | 0.00212 | -0.14822 |
| Glucose (avg) | 0.00208 | 0.10525 |
| QTc interval | 0.00207 | -0.08236 |
| BNP missing | 0.00195 | -0.05069 |
| Depression diagnosis | 0.00186 | -0.23337 |
| Non-dihydropyridine calcium channel blocker | 0.00176 | -0.45687 |
| Alpha agonist | 0.00173 | 0.06710 |
| Ivabradine | 0.00155 | 0.24713 |
| Black race | 0.00153 | 0.00364 |
| Thiazide | 0.00153 | -0.50378 |
| Hemoglobin A1C 6.5-7.5% | 0.00140 | -0.17976 |
| Other race | 0.00139 | -0.27647 |
| Insurance type: Other | 0.00138 | -0.77486 |
| COPD | 0.00123 | -0.44836 |
| NTproBNP ≥ 1000 ng/L | 0.00115 | 0.19448 |
| BNP >250 ng/L | 0.00111 | 0.25017 |
| Sulfonylurea | 0.00094 | 0.03557 |
| Sotalol | 0.00094 | 0.22396 |
| BNP ≤ 250 ng/L | 0.00082 | -0.11649 |
| Non-Hispanic ethnicity | 0.00080 | 0.16881 |
| Hispanic ethnicity | 0.00080 | 0.08916 |
| Clonidine | 0.00074 | 0.25595 |
| Hypertension | 0.00073 | -0.13465 |
| Asthma phenotype | 0.00070 | -0.33963 |
| Relationship status: Other | 0.00069 | -0.22746 |
| NTproBNP < 1000 ng/L | 0.00067 | 0.14391 |
| Hemoglobin A1C ≥ 7.5% | 0.00044 | -0.24069 |
| Social security number | 0.00044 | -0.61146 |
| SGLT2 inhibitor | 0.00037 | 0.18302 |
| Anxiety diagnosis | 0.00034 | 0.10978 |
| Prasugrel | 0.00029 | 0.30607 |
| Nifedipine | 0.00028 | 0.40787 |
| Glucagon-like peptide-1 receptor agonist | 0.00025 | -0.35253 |
| Sodium polystyrene | 0.00025 | 0.17042 |
| COPD phenotype | 0.00024 | -0.04108 |
| Ezetimibe | 0.00023 | -0.04552 |
| Insurance type: Self pay | 0.00021 | 0.18958 |
| Cardiac resynchronization therapy | 0.00021 | -0.01746 |
| Language: English | 0.00020 | 0.69344 |
| Ventricular assist device | 0.00016 | -0.46493 |
| Interpreter needed | 0.00015 | -0.44213 |
| PCSK9 inhibitor | 0.00014 | 0.16254 |
| Language: Spanish | 0.00013 | -0.54113 |
| Other ethnicity | 0.00009 | -0.24637 |
| Thiazolidinedione | 0.00006 | 0.00301 |
| Unknown race | 0.00006 | -0.58112 |
| Patiromer | 0.00005 | 0.31449 |
| Language: Other/Unknown | 0.00004 | -0.11894 |
| Angioedema | 0.00001 | -0.57025 |
| Calcium 8.4-10.6 mg/dL | 0.00001 | -0.66995 |
| ACE-induced angioedema | 0.00000 | -0.55846 |
| Cilostazol | 0.00000 | -0.56036 |
| Sodium zirconium | 0.00000 | -0.56303 |
| Calcium > 10.6 mg/dL | 0.00000 | 0.00000 |
| Digoxin | 0.00000 | 0.00000 |
| Aliskiren | 0.00000 | 0.00000 |

| **Random forest features of importance for ACE/ARB prescribing** | **SHAP Value** | **Correlation Coefficient** |
| --- | --- | --- |
| Evidence-based BB | 0.06216 | 0.92348 |
| MRA | 0.03496 | 0.86599 |
| Aspirin | 0.03371 | 0.86789 |
| Statin | 0.02521 | 0.78201 |
| Loop Diuretic | 0.02097 | 0.78531 |
| # Unique medications | 0.02093 | 0.61028 |
| eGFR | 0.01608 | 0.59234 |
| Age | 0.01599 | -0.47249 |
| Serum Creatinine | 0.01350 | -0.45027 |
| Ejection Fraction | 0.01110 | -0.52491 |
| K supplement | 0.01092 | 0.74311 |
| Glucose | 0.01081 | -0.32021 |
| ARNI | 0.01026 | 0.92520 |
| Glucose (avg) | 0.00909 | -0.49230 |
| Potassium | 0.00902 | 0.66940 |
| Hemoglobin | 0.00886 | 0.55488 |
| BUN | 0.00845 | -0.57866 |
| Albumin | 0.00765 | -0.00861 |
| Non-EBM BB | 0.00746 | 0.71246 |
| Sodium | 0.00715 | -0.52564 |
| Systolic BP | 0.00679 | -0.10373 |
| Metformin | 0.00667 | 0.80007 |
| Systolic BP (avg) | 0.00664 | -0.21345 |
| QRS Interval | 0.00653 | 0.29453 |
| Non-dihydropyridine calcium channel blocker | 0.00644 | 0.81731 |
| Weight | 0.00631 | 0.36651 |
| Prothrombin time | 0.00631 | -0.28591 |
| ALT | 0.00621 | 0.25219 |
| Heart Rate (avg) | 0.00615 | -0.30191 |
| International normalized ratio | 0.00588 | -0.21100 |
| BMI | 0.00587 | 0.20908 |
| QTc Interval | 0.00585 | -0.20374 |
| Diastolic BP (avg) | 0.00574 | -0.00032 |
| Height | 0.00561 | 0.05758 |
| Heart Rate | 0.00551 | -0.38584 |
| Diastolic BP | 0.00542 | 0.00116 |
| Hypertension | 0.00529 | 0.79406 |
| Bilirubin | 0.00522 | -0.11652 |
| Inhaled anticholinergic | 0.00461 | 0.63161 |
| Metoprolol tartrate | 0.00446 | 0.69921 |
| Respiratory Rate | 0.00366 | -0.32553 |
| Myocardial Infarction | 0.00333 | 0.54611 |
| Thiazide | 0.00307 | 0.59160 |
| Insulin | 0.00301 | 0.42082 |
| Clopidogrel | 0.00270 | 0.56883 |
| CKD | 0.00268 | -0.58252 |
| DOAC | 0.00258 | 0.48853 |
| Coronary artery disease | 0.00252 | 0.58223 |
| Hemoglobin A1C missing | 0.00240 | -0.61612 |
| Atrial Fibrillation | 0.00224 | -0.64589 |
| Insurance type: Medicare/Tricare | 0.00218 | -0.53824 |
| ICD | 0.00217 | 0.51218 |
| Nonischemic cardiomyopathy | 0.00204 | -0.57093 |
| Diabetes | 0.00187 | 0.67124 |
| Warfarin | 0.00179 | 0.28448 |
| COPD | 0.00147 | -0.66863 |
| Tobacco Use | 0.00143 | 0.47045 |
| Relationship status: In Relationship | 0.00142 | 0.50553 |
| Insurance type: Medicaid/Dual | 0.00134 | 0.61287 |
| White race | 0.00133 | -0.57468 |
| Hemoglobin A1C < 6.5% | 0.00128 | 0.42780 |
| Asthma | 0.00123 | -0.41830 |
| Calcium missing | 0.00115 | 0.66639 |
| Relationship status: Single | 0.00113 | 0.02553 |
| Relationship status: Other | 0.00113 | -0.76102 |
| Insurance type: Other | 0.00112 | -0.78961 |
| Hypotension | 0.00108 | -0.59127 |
| Microalbumin missing | 0.00107 | -0.37629 |
| Amlodipine | 0.00105 | 0.31940 |
| Alcohol use | 0.00100 | 0.32357 |
| Alpha agonist | 0.00097 | -0.49816 |
| Female | 0.00096 | -0.21861 |
| NTproBNP missing | 0.00096 | 0.67445 |
| BNP >250 ng/L | 0.00093 | -0.75220 |
| Hemoglobin A1C 6.5-7.5% | 0.00090 | -0.71432 |
| Black race | 0.00089 | 0.53139 |
| Calcium < 8.4 mg/dL | 0.00088 | -0.59064 |
| BNP missing | 0.00085 | 0.69039 |
| Angioedema | 0.00082 | -0.83089 |
| Insurance type: Self pay | 0.00074 | -0.82033 |
| COPD | 0.00074 | 0.22574 |
| NTproBNP ≥ 1000 ng/L | 0.00072 | -0.76735 |
| Ezetimibe | 0.00070 | -0.74805 |
| Depression diagnosis | 0.00069 | -0.61198 |
| NTproBNP < 1000 ng/L | 0.00066 | -0.58133 |
| Unknown race | 0.00059 | -0.73171 |
| Insurance type: commercial | 0.00058 | 0.10656 |
| Right heart catheterization | 0.00057 | -0.69813 |
| Hemoglobin A1C ≥ 7.5% | 0.00057 | -0.07277 |
| Non-Hispanic ethnicity | 0.00056 | 0.47383 |
| Hyperkalemia | 0.00055 | 0.10518 |
| Asthma phenotype | 0.00054 | -0.62299 |
| Hispanic ethnicity | 0.00052 | -0.35993 |
| Other race | 0.00047 | -0.11601 |
| Microalbumin ≥ 30 mg/dL | 0.00046 | 0.41126 |
| Other ethnicity | 0.00045 | -0.74331 |
| Microalbumin < 30 mg/dL | 0.00044 | -0.17290 |
| Sotalol | 0.00043 | -0.54880 |
| Sulfonylurea | 0.00043 | 0.14245 |
| Social security number | 0.00037 | -0.40765 |
| Anxiety diagnosis | 0.00032 | -0.74669 |
| Ticagrelor | 0.00032 | 0.56882 |
| BNP ≤ 250 ng/L | 0.00032 | -0.64797 |
| Language: English | 0.00026 | 0.30199 |
| Ventricular assist device | 0.00024 | 0.25023 |
| Language: Spanish | 0.00023 | -0.18912 |
| COPD phenotype | 0.00023 | -0.64654 |
| Interpreter needed | 0.00022 | -0.48567 |
| Prasugrel | 0.00020 | -0.76789 |
| Glucagon-like peptide-1 receptor agonist | 0.00020 | -0.65583 |
| ACE-induced angioedema | 0.00018 | -0.76128 |
| Language: Other/Unknown | 0.00018 | -0.55408 |
| Clonidine | 0.00017 | -0.07604 |
| SGLT2 inhibitor | 0.00017 | -0.41487 |
| Cardiac resynchronization therapy | 0.00016 | -0.65978 |
| Sodium polystyrene | 0.00014 | -0.02998 |
| Thiazolidinedione | 0.00009 | -0.53111 |
| PCSK9 Inhibitor | 0.00006 | -0.58503 |
| Cilostazol | 0.00006 | -0.64771 |
| Ivabradine | 0.00006 | 0.30394 |
| Calcium > 10.6 mg/dL | 0.00004 | -0.88768 |
| Nifedipine | 0.00003 | 0.57349 |
| Calcium 8.4-10.6 mg/dL | 0.00001 | 0.63735 |
| Digoxin | 0.00001 | 0.69620 |
| Patiromer | 0.00000 | 0.86247 |
| Sodium zirconium | 0.00000 | 0.00000 |
| Aliskiren | 0.00000 | 0.00000 |

Values are most recent values unless denoted as “average.”
